# 

**Published:** 1911-12-23

**Authors:** 


### Florence Nightingale Memorial.

We have received the following contributions since those acknowledged last week. They are for a statue, unless otherwise indicated :—

Right Hon. Lord Cowdray, £20; Sir James Goodhart, Bart., £3 3s.; Sir William Clayton, Bart., £1 1s.; Henry Howorth, Esq., 10s; Miss F. E. Ball (for annuities), 7s. 6d.

Received by Mr. G. Q. Roberts, Hon. Secretary :—The Worshipful Company of Goldsmiths, £100; collected by Miss Anderson, matron, Colonial Hospital, Fiji, £88 18s. 3d. (this is a record of splendid enthusiasm—a collection of small sums varying from 3d. to £5 from all the scattered islands of Fiji); The Worshipful Company of Drapers, £26 5s.; The Worshipful Company of Salters, £10 10s.; Miss Warren, £10 10s.; Sir Walpole Greenwell, Bart., £5 5s.; Robinson & Sons, Limited, Chesterfield, £2; Vice-Admiral S. C. Colville, £2; 2nd Battalion York and Lancaster Regiment, £2; Right Hon. Louis Harcourt, £2; Miss A. F. Ferryman, £1 1s.; collection per Mrs. Arthur Wagg, £1 1s.; Mrs. M. E. Crewe, £1 1s.; "Two Sisters," £1 1s.; nursing staff, Isolation Hospital, Leyton, £1; Miss A. Vesey Fitzgerald, £1; Miss E. Fremlin, £1; Miss M. E. Tabor, £1; girls of the Nightingale House, Secondary School, Castleford, 15s. 6d.; Miss Rogers, Leicester Infirmary, 10s. 6d.; nursing staff, Royal Southern Hospital, Liverpool, 10s.; Mrs. William Best, 5s.; Miss F. Epton, 3s. 6d.; "J. H.," 2s. 6d.; Mrs. E. D. Fenning, 2s.

Further contributions may be sent to the Hon. Secretary, Florence Nightingale Memorial, St. Thomas' Hospital, Albert Embankment, London, S.E.; or to the Editor of The Hospital, 29 Southampton Street, Strand, London, W.C.

---

The Queen has sent a Christmas donation of £10 to Queen Charlotte's Hospital, of which she is patron.

### Appointments.

Dr. Norman Maclaren, who has been for seven years assistant surgeon to Cumberland Infirmary, has been reappointed.

Mr. Frederick Pybus, M.S., F.R.C.S., has been appointed surgeon to the Hospital for Sick Children, Newcastle-on-Tyne.

Mr. J. Leishman, the Edinburgh City Treasurer, has been appointed chairman of the Scottish Commissioners under the Insurance Act, and Dr. J. Christie McVail deputy-chairman.

Mr. J. P. Musson, M.B., Ch.B. honours (Leeds), late senior house surgeon at the Hospital for Women and Children, Leeds, has been appointed junior house surgeon to Beckett Hospital, Barnsley. Mr. Musson was trained at the Leeds Infirmary Medical School and General Infirmary.

At the December meeting of the Board the "best thanks" of the Bradford Royal Infirmary have been given to Mr. J. Basil Mall, M.C., Cantab., for the services he has rendered as honorory surgeon for the past ten years, and he has been reappointed honorary surgeon for another term of ten years; and to Mr. J. Beattie Dunlop, M.B., Cantab., for the services he has rendered as honorary assistant physician for the past five years, to which he has been reappointed for a second similar term.

### The Week's Novelties.

### A NEW TOBACCO FOR C[illegible]MAS.

All Institutional workers who smok[illegible] CHRIST[illegible] hours should try a curious new tobacco, [illegible]e in off-du[illegible] the Voltuma Company, 22 Minories, London, [illegible]atented by [illegible] manufactures electrified or ozonised cigars an[illegible] E.C., which These novel "smokes" should be tested by [illegible]d cigarettes. the non-electrified with the electrified Virginia c[illegible] comparing which the Company makes from the same tobac[illegible] igarettes, packs in the same sample box. Soft, sweet, and m[illegible]o, and are the qualities which the electrified cigarette possesses[illegible]ellow in a greater degree than its unimproved fellow. The inventor, Mr. E. J. Liesby, claims that the electrification modifies the nicotine, and consequently is less injurious to inhalers. Hospital officers are thus able to offer a new cigarette, than which is no more popular Christmas present.

### JAEGER SARDINES.

(The Jaeger Smedsvik Company, Naugesund, Norway; London address: Chesterfield House, 98 Great Tower Street, E.C.)

As an article of diet the sardine is one of the most valuable items on the breakfast menu, not, be it added, served on toast in the dry way which is unfortunately so common, but fresh or from the tin of a reputable manufacturer. It contains a large percentage of easily assimilable proteid, and an equally large amount of fat in a form which experience has shown is the most absorbable. The Jaeger brand of sardines, which we have recently tried, is one of the best of the numerous varieties in the market. The fish are small, the bones well "steeped," and the flesh of a delicate flavour and perfect flakiness. The olive oil in which they are preserved is of good taste and free from any suspicion of rancidness, while the tomato sauce—for this brand is of the kind known as "Tomato sardines"—is equally excellent. The low price at which these sardines are sold should make them popular, especially in view of the fact that they are superior in some ways to many much more expensive brands.

### DE KUYPER'S HOLLANDS.

(John De Kuyper and Sons, Rotterdam.)

The sample of "Geneva" which we have received from this well-known and historical firm may honestly be said to merit the claims which are made for the special brand of gin known all the world over as De Kuyper's Hollands. It is a pure malt spirit, flavoured with juniper, and possessing a pleasant, spirituous, bland, yet stimulating, taste, while its aroma is distinctly superior to that of most gins on the market. This, we hold, is due to the care taken in distillation. The juniper berries are distilled with the original malt, so that the volatile ethereal oils are incorporated in the spirit, and not merely part of them added, as is done when gins are flavoured by saturation or percolation methods. The "Geneva" is a dry brand, and particularly suitable to those who desire a moderately high alcoholic liqueur. Gin is a valuable diuretic, and its medicinal properties should be better recognised by the profession than they appear to be. Those who wish to test them can do no better than make the experiment with De Kuyper's Hollands, which is, perhaps, the best and purest variety of gin obtainable.

---

Gifts of pheasants from Lord Barnard, Colonel Curre and Mrs. H. J. Allcroft have been received for the use of the patients of the Royal Hospital for Incurables, Putney Heath.

